# Anti-Inflammatory and Chondroprotective Effects Induced by Phenolic Compounds from Onion Waste Extracts in ATDC-5 Chondrogenic Cell Line

**DOI:** 10.3390/antiox11122381

**Published:** 2022-12-01

**Authors:** Mónica Paesa, Carmen Ancín-Azpilicueta, Gustavo Velderrain-Rodríguez, Olga Martin-Belloso, Oreste Gualillo, Jesús Osada, Maria Jesús Rodríguez-Yoldi, Gracia Mendoza

**Affiliations:** 1 Department of Chemical Engineering, University of Zaragoza, Campus Río Ebro-Edificio I+D, C/ Poeta Mariano Esquillor S/N, 50009 Zaragoza, Spain; 2 Department of Sciences, Institute for Advanced Materials (INAMAT2), Navarra Public University, Campus Arrosadía s/n, 31006 Pamplona, Spain; 3 Alianza Latinoamericana De Nutricion Responsable, Inc., 400 E Randolph St Suite 2305, Chicago, IL 60611, USA; 4 Department of Food Technology, University of Lleida—Agrotecnio Center, Av. Alcalde Rovira Roure 191, 25198 Lleida, Spain; 5 SERGAS (Servizo Galego de Saude) and IDIS (Instituto de Investigación Sanitaria de Santiago de Compostela), NEIRID Lab (Neuroendocrine Interactions in Rheumatology and Inflammatory Diseases), Research Laboratory 9, Santiago University Clinical Hospital, 15706 Santiago de Compostela, Spain; 6 Department of Biochemistry and Molecular Cell Biology, Veterinary Faculty, Health Research Institute of Aragon-University of Zaragoza, 50009 Zaragoza, Spain; 7 CIBER de Fisiopatología de la Obesidad y la Nutrición, Instituto de Salud Carlos III, 28029 Madrid, Spain; 8 Instituto Agroalimentario de Aragón, CITA-Universidad de Zaragoza, 50009 Zaragoza, Spain; 9 Department of Pharmacology and Physiology, Forensic and Legal Medicine, Veterinary Faculty, Health Research Institute of Aragon-University of Zaragoza, 50009 Zaragoza, Spain; 10 Aragon Health Research Institute (IIS Aragon), 50009 Zaragoza, Spain

**Keywords:** *Allium cepa* L. waste extracts, circular economy, osteoarthritis, inflammation, ROS, NF-κB, chondroprotective

## Abstract

Osteoarthritis is a prevalent degenerative condition that is closely related to the destruction and inflammation of cartilage. The high prevalence of this pathology exhorts researchers to search for novel therapeutic approaches. Vegetable–fruit wastes have emerged as a promising origin of anti-inflammatory and antioxidant compounds that, in some cases, may also exert chondroprotective effects. This study aims to decipher the potential of onion waste products in the inhibition of molecular events involved in osteoarthritis. Onion extracts showed a high content of phenolic compounds and antioxidant properties. Cytocompatibility was demonstrated in the chondrogenic cell line ATDC-5, exerting viability percentages higher than 90% and a slight increase in the S phase cycle cell. The induction of inflammation mediated by the lipopolysaccharide and onion extracts’ treatment substantially inhibited molecular markers related to inflammation and cartilage degradation, highlighting the promising application of onion extracts in biomedical approaches. The in silico analyses suggested that the results could be attributed to protocatechuic, ellagic, and vanillic acids’ greater cell membrane permeability. Our work provides distinctive information about the possible application of waste onion extracts as functional components with anti-inflammatory and chondroprotective characteristics in osteoarthritis.

## 1. Introduction

Osteoarthritis (OA) affects more than 500 million individuals worldwide, being the leading cause of pain and functional disability among musculoskeletal diseases. In addition to mechanical derangement and cartilage degradation, inflammation is crucial in OA development and progression, together with the subsequent remodeling of the adjacent bone. The increase in life expectancy together with current changes regarding sedentary lifestyles and obesity point to a higher prevalence of this pathology. In fact, it is anticipated that by 2030, the prevalence of OA will substantially rise, reaching 25–30% in different countries [1,2,3].

The hallmark of OA is cartilage degradation, which implies a change in chondrocytes phenotype, the production of proteolytic enzymes, and the synthesis of inflammatory mediators [4]. Pathogen-related chemical patterns (PAMPs), such as lipopolysaccharides (LPS), can be recognized by Toll-like receptors (TLRs). In particular, TLR4 was specifically associated with OA due to its capacity to trigger inflammatory responses in cartilage and cartilage breakdown [5]. Cartilage catabolism includes extracellular matrix (ECM) degradation, triggering the expression of molecular markers such as matrix metalloproteinases (MMP) 3 and 13, together with A disintegrin and metalloproteinase with thrombospondin motifs-4,5 (ADAMTS-4, ADAMTS-5), among others. Nitric oxide (NO), reactive oxygen species (ROS), and concomitant oxidative stress are all produced after the activation of inflammation-induced pathways, leading to the overexpression of molecular markers such as inducible nitric oxide synthase (iNOS) and several cytokines (such as interleukin (IL) 1, 6, and 8) [6]. In addition, one of the mechanisms contributing to the development of OA is an aberrant activation of the nuclear factor kappa-light-chain-enhancer of activated B cells (NF-κB). This factor is then activated by cytokines and the metabolites obtained from ECM breakdown, which leads to the activation of IκB kinase (IKK), the phosphorylation of the NF-B p65 subunit, and its translocation to the nucleus [7]. Therefore, blocking the LPS/TLR4/NF-B complex may be a potential target for treating OA [8,9].

Several studies have emphasized the relevant role of different antioxidant and anti-inflammatory compounds in alleviating cartilage damage [10,11,12,13,14,15,16,17,18]. In this regard, the search for novel products with antioxidant and anti-inflammatory abilities is appealing to develop more effective strategies in OA treatment. Natural products obtained from the valorization of different vegetables and fruits have become very interesting as a source of biomolecules. Specifically, onion (*Allium cepa* L.) bulb crops have increased in the last years as a result of its interest as a food condiment. As a consequence, derived residues are largely produced worldwide with the subsequent environmental impact. With this scenario, the valorization of onion waste points out to be beneficial not only for the reduction of the environmental impact but also to yield bioactive molecules. These onion waste and by-products are rich in flavonoids, yielding 0.03–1 g/kg in the edible part and up to 10 g/kg in the skin [19,20,21]. Flavonols are the main beneficial compounds present in onions with more than 25 types identified. Among them, quercetin and its derivatives, being well-known inhibitors of oxidative damage, are particularly abundant in onions [22,23,24,25]. Flavonoids were also shown to be effective molecules against MMP-mediated damage in chondrocytes [10]. Moreover, the content of flavonoids depends on onion variety, geographical area of cultivation, storage, and processing, resulting in alterations in biomolecules’ structure and bioavailability [26,27].

The main objective of the current study was to assess the chondroprotective and anti-inflammatory benefits of onion extracts (OE) in a chondrogenic cell line as a novel approach in the treatment of OA. Cellular and molecular events were studied to decipher the feasibility of OE in OA therapy.

## 2. Materials and Methods

### 2.1. Extracts Obtaining and Phenolic Compound Characterization

Onion extracts and their characterization regarding the presence of phenolic compounds were developed as we previously described [28]. In brief, onion extract from household waste was obtained by drying at 30 °C and milling through a coffee grinder to sieve the material by using a 300 µm sieve. The extractions were carried out using 100 mL of an ethanol:water solution (50:50 *v:v*) and 1 g of onion waste powder and incubation under shaking (250 rpm) at 40 °C for 24 h. Then, onion extracts were obtained by centrifugation (8586 g, 15 min), filtration, rotoevaporation, and further lyophilization to finally obtain an extraction yield of 20%.

The phenolic components in the extracts were characterized by HPLC-DAD as described before [28,29]. In brief, 350 mL of methanol was used to reconstitute 30 mg of freeze-dried extract. This mixture was then filtered using a 0.45 μm PTFE syringe filter. Samples were then analyzed by HPLC (Waters Div., Milford, MA, USA). Phenolic compounds were identified and quantified by means of a calibration curve for each of the compounds (R^2^ ≥ 0.99). The extract composition was expressed in mg/g of dry extract.

### 2.2. Onion Extracts Antioxidant Properties

As previously reported [28,30,31], three spectrophotometric techniques were employed to assess the antioxidant capacity of onion extracts. In brief, the DPPH (2,2-diphenyl-1-pycrilhydracyl) method was carried out by dissolving the extracts (49.1–53.1 mg) in methanol (5 mL), filtering, and then diluting them 10 times in methanol. The ABTS (2,20-azinobis(3-ethylbenzothiazoline-6-sulphonic acid)) [30] and the FRAP [31] assays were performed as published [28,30,31]. Trolox (0.05–1.2 mM) was used as a standard in the three spectrophotometric methods (R^2^ = 0.99). The determinations were carried out in a UV-Vis spectrophotometer (Jenway 7315, Staffordshire, UK). Three different processed samples were used in all the methods, and the antioxidant activity was represented as mmol Trolox equivalents (TE)/g of dry extract.

### 2.3. Quantification of Total Phenolic and Flavonoid Content

Total phenolic and flavonoid quantification was carried out as previously described [28,32,33]. Briefly, 50 mg of the extract was dissolved in 5 mL of methanol and filtered to be further diluted in methanol (4 times for total phenolic content and 30 times for total flavonoid quantification). The Folin-Ciocalteu method was used to quantify the total phenolic content [32], whereas a colorimetric assay based on the interaction of flavonoids with AlCl_3_ in acetic acid was developed [33]. Gallic acid (0.2–5.1 mM) and quercetin (3–30 µg/mL) were used as standards in the total phenolic and flavonoid content determinations, respectively (R^2^ = 0.99). The determinations were carried out in a UV-Vis spectrophotometer (Jenway 7315, Staffordshire, UK) at 765 nm for total phenolic content and at 420 nm to quantify the flavonoid content. Three different processed samples were used and mg of gallic acid or quercetin/g of dry extract were used to express the results.

### 2.4. Cell Culture and Viability

The mouse chondrogenic cell line ATDC-5 was kindly gifted by Dr. Oreste Gualillo (University of Santiago de Compostela, La Coruña, Spain). Cells were cultivated in Dulbecco’s Modified Eagle’s Medium (DMEM)–Ham’s F-12 medium (Biowest, Nuaillé, France) supplemented with 5% FBS (Gibco, UK), 10 μg/mL human transferrin (Sigma-Aldrich, St. Louis, USA), 3 × 10^−8^ M sodium selenite (Sigma-Aldrich, St. Louis, MO, USA), 1% penicillin-streptomycin-amphotericin B (Biowest, Nuaillé, France), and 1% stable glutamine (Biowest, Nuaillé, France) in a humidified atmosphere of 5% CO_2_ at 37 °C.

The viability of cells was assessed using the Blue Cell Viability Assay (Abnova, Taipei, Taiwan). In brief, cells (10^4^ cells/well) were cultured in 96-well plates and treated with onion extracts (OE) (1.9–62.5 µg/mL) for 24 h at 37 °C. After that, the reagent was added in accordance with the manufacturer’s instructions (10%; incubation of 4 h at 37 °C and 5% CO_2_). Metabolically active cells were able to reduce the dye, and the fluorescence generated was calculated in a microplate reader (Varioskan Lux, Thermo Fisher, Waltham, MA, USA) at 530 nm excitation and 590 nm emission wavelengths. Viability percentages were determined by linear interpolation of data considering control samples (non-treated cells) as 100% of viability.

### 2.5. Analysis of Cell Cycle and DNA Content

The influence of OE on the ATDC-5 cell cycle was studied using the PI/RNase solution kit (Immunostep, Salamanca, Spain). Briefly, cells (5.7 × 10^4^ cells/cm^2^) were cultured in 24-well plates and treated with the subcytotoxic concentration of OE. After that, cells were fixed in 70% ice-cold ethanol and kept at 4 °C for 48 h. Samples were then centrifuged, washed in PBS, and stained with a propidium iodide (PI) solution (50 μg/mL) that contained 100 μg/L of RNase A. By using a Beckman Coulter Gallios cytometer (Brea, CA, USA), the DNA content of the PI-stained cells was determined by flow cytometry. At least three separate tests were carried out in triplicate.

### 2.6. Evaluation of Cell Apoptosis

Cells were seeded in 12-well plates (5.7 × 10^4^ cell/cm^2^) and treated with OE for 24 h at the highest concentration assayed (62.5 µg/mL). Then, cells were harvested and stained with an annexin V-FITC and propidium iodide kit (Immunostep, Salamanca, Spain), as previously described [34]. After incubation, cells were resuspended in 100 µL of Annexin V Binding Buffer (100 mM HEPES/NaOH pH 7.4, 140 mM NaCl, 2.5 mM CaCl_2_), 5 µL of Annexin V-FITC, and 5 µL of propidium iodide. Then, 400 µL of Annexin Binding Buffer was added after 15 min of incubation at room temperature in the dark. By using a Beckman Coulter Gallios (Brea, CA, USA), the signal intensity was quantified. Three separate studies were performed in triplicate to calculate the percentage of apoptosis.

### 2.7. Determination of Intracellular Levels of Reactive Oxygen Species (ROS)

As previously mentioned [34], the dichlorofluorescein test was used to measure the intracellular amount of ROS. In brief, cells were grown in 96-well plates at a density of 10^4^ cells/well and exposed to OE (1.9–62.5 µg/mL) for 24 h. After incubation, 20 µM of 20,70– dichlorofluorescein diacetate (DCFH-DA; D6883; Sigma-Aldrich, St. Louis, MO, USA) was added to the cells for 20 min in PBS at 37 °C. Using a microplate reader (Varioskan Lux, Thermo Fisher, Waltham, MA, USA) and emission and excitation wavelengths of 535 nm and 485 nm, respectively, the fluorescence produced by the oxidation of DCF was measured. A fold change vs. control was used to express the ROS levels. Data were collected in triplicate from three separate studies.

### 2.8. LPS Treatment and Nitrite Assay

In vitro induction of inflammation was set up to assess the potential anti-inflammatory effects of OE. ATDC-5 cells were cultured at a density of 5.7 × 10^4^ cells/cm^2^ for 24 h. After that, cells were treated with 250 ng/mL of lipopolysaccharide (LPS; Serotype O55:B5; Sigma-Aldrich, St. Louis, MO, USA) for 24 h at 37 °C after being pre-incubated with OE (1.9–62.5 g/mL) in serum-free media for 4 h at 37 °C. The Griess reaction was used to assess nitrite accumulation in culture media. In brief, 100 μL of cell culture media and 100 μL of Griess reagent (Sigma-Aldrich, St. Louis, MO, USA) were mixed and incubated for 15 min at room temperature. The absorbance was measured at 540 nm using a microplate reader (Varioskan Lux, Thermo Fisher, Waltham, MA, USA). At least three separate experiments were carried out in triplicate.

### 2.9. mRNA Expression Level Analyses

After treatment with OE and/or stimulation with LPS, ATDC-5 cells were collected to evaluate the expression of inflammation and cartilage degradation genes by quantitative reverse transcription PCR (RT-qPCR). PBS and TRIzol (ThermoFisher, Waltham, MA, USA) were used to wash and lyse the cells. RNA levels were measured in NanoDrop 2000 (Thermo Fisher Scientific, Waltham, MA, USA). Using the PrimeScript™ RT Master Mix (Takara Bio, Shiga, Japan) and 500 ng of total RNA, first-strand cDNA was synthesized. The amplification was carried out using QuantStudioTM 5 Real-Time PCR Instrument (Applied Biosystems, Waltham, MA, USA) under the following conditions: 30 s at 95 °C, followed by 40 cycles of 5 s at 95 °C and 30 s at 60 °C. The reaction was performed in a total volume of 20 μL containing 2 μL diluted cDNA, 2 μL primers, 10 µL MasterMix (Takara Bio, Shiga, Japan), 5.64 μL sterile purified water, and 0.36 μL of ROX reference dye II (Takara Bio, Shiga, Japan). The following target probes were used to assess gene expression (Integrated DNA Technologies, Neward, NJ, USA): Mouse *Nos2* (Mm.PT.58.43705194), *Mmp13* (Mm.PT.58.42286812), *Mmp3* (Mm.PT.58.9719290), *Il6* (Mm.PT.58.10005566), *Il8* (Mm.PT.58.9981538), *Cxcl1* (Mm.PT.58.42076891), and *Adamtl4* (Mm.PT.58.11009098). The level of the target mRNA was normalized to the level of *Actb* (Mm.PT.39a.22214843.g) and expressed as a percentage of LPS, which was arbitrarily set to 100%. The 2^−ΔΔCT^ method was used to analyze the data. For each gene, triplicates of three different experiments were carried out.

### 2.10. Immunofluorescence

Cells were grown at a density of 5.7 × 10^4^ cells/cm^2^ onto 24-well plates on glass coverslips. After treatment with OE and/or stimulation with LPS, cells were fixed for 15 min with freshly cold formaldehyde (3.7%). Then, cells were washed with PBS and incubated with blocking buffer (which contains 5% normal goat serum (Sigma-Aldrich, St. Louis, MO, USA) and 0.3% Triton X-100 (Sigma-Aldrich, St. Louis, MO, USA)) for 1 h at room temperature. Next, primary anti-NF-κB/p65 rabbit monoclonal antibody (1:100; Abcam, Cambridge, UK) was added to each well and incubated at 4 °C overnight. After this, cells were incubated with secondary Alexa Fluor^®^ 488 goat anti-rabbit IgG (H + L) polyclonal antibody (1:1000; Molecular Probes, Eugene, OR, USA) for 2 h and stained with 2 µg/mL of DAPI (Molecular Probes, Eugene, OR, USA) for 10 min at room temperature [35]. Mowiol mounting medium (Thermo Fisher, Waltham, MA, USA) was used to attach coverslips to glass slides. Confocal microscopy (Leica TCS SP2 Laser Scanning Confocal Microscope, München, Germany) was used to visualize the samples. Three separate experiments were conducted.

### 2.11. Cell Permeability and Pharmacokinetic Properties of Phenolic Compounds

The passive cellular permeability and pharmacokinetic properties of the major individual phenolic molecules present in OE were studied as reported before [36]. These properties were obtained by an in silico analysis using the SMILES codification and the pkCSM program (http://biosig.unimelb.edu.au/pkcsm/prediction, accessed on 21 November 2022). These codes served as a description of the molecular structure of these compounds and could be easily obtained from the PubChemOpen Chemistry Database (https://pubchem.ncbi.nlm.nih.gov/search/, accessed on 3 November 2022). Finally, the cell permeability of these molecules was also predicted, and is expressed as the log P_app_ and expressed as 10^−6^ cm/s.

### 2.12. Statistical Analyses

Statistical analyses of the data were performed using GraphPad version 7.04 software (GraphPad Software, San Diego, CA, USA). The normal distribution of the variables was analyzed by the Shapiro−Wilk test followed by a one-way analysis of variance (ANOVA) set for multiple comparisons with Bonferroni–Sidak’s post-hoc tests. All tests were performed at least three times. Data are presented as mean ± SD.

## 3. Results and Discussion

### 3.1. Antioxidant Properties and Phenolic Content of Onion Extracts

Onion extracts from the same origin as those tested in a recent work carried out by the group about the therapeutic use of these residues on colon cancer [28] were used. The composition of the extracts for which the described results were obtained is listed below. The antioxidant capacity of the onion extracts by ABTS method provided the highest values (1.11 ± 0.08 mmol Trolox/g extract), whereas the lowest values were obtained with DPPH (0.49 ± 0.08 mmol Trolox/g extract) and FRAP (0.83 ± 0.01 mmol Trolox/g extract). According to the differences between the three techniques, these compounds may be working on various antioxidant processes depending on the chemical environment and the structure of free radicals. The total phenolic content of onion extracts was 177 ± 9 mg of gallic acid/g extract and the total flavonoid content was 64 ± 3 mg of quercetin/g extract [28]. In total, seven different phenolic compounds were identified in the onion extract obtained from onion waste. Three of them (protocatechuic acid 11.5 ± 0.3 mg/g extract, vanillic acid 0.33 ± 0.03 mg/g extract and ellagic acid 0.10 ± 0.01 mg/g extract) are phenolic acids, and the rest are flavonols, with quercetin being one of the main components (10.2 ± 0.4 mg/g extract).

### 3.2. Cellular and Molecular Effects of Onion Extracts in ATDC-5 Cells

The role of natural compounds in OA and their potential effects on chondroprotection have been linked to their antioxidant and anti-inflammatory ability to prevent cartilage degeneration [7,37,38]. Phenolic compounds and specifically flavonoids have been widely reported as efficient inhibitors of catabolic processes in chondrocytes depleting the expression of characteristic molecular markers such as NO, iNOS, MMP-3, MMP-13, ADAMTS-4, IL-1β, IL-6, and IL-8, among others [7]. The anti-inflammatory effects of these compounds have a great impact on ECM degradation processes owing to their capability to alleviate the inhibition of matrix synthesis and the activation of proteinases activity [16]. Our studies have focused on these effects to evaluate the potential of OE in the protection of chondrocytes from inflammation and degeneration.

#### 3.2.1. Cellular Effects and Intracellular ROS Levels after Onion Extracts Treatment

**Figure 1:**
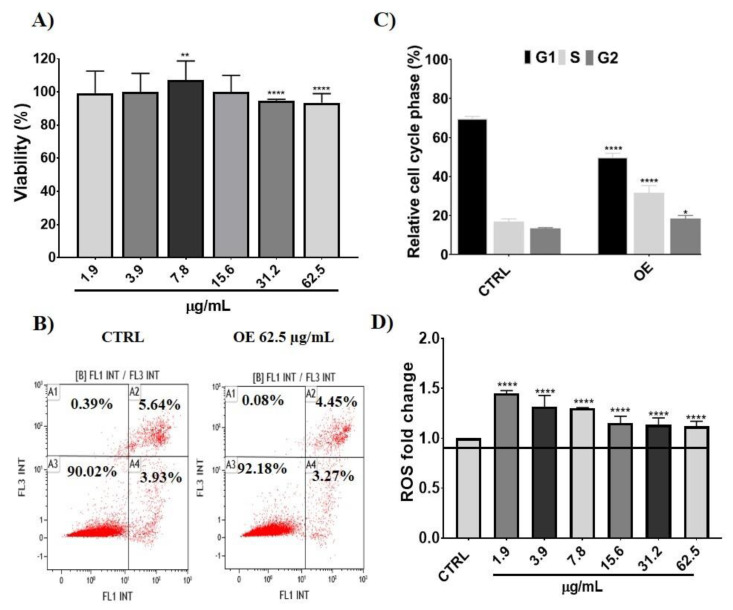
Effect of onion extracts on ATDC-5 cells. (**A**) Cell viability after treatment with different onion extract concentrations during 24 h using the Blue Cell Viability Assay. Control sample (untreated cells) = 100% viability. (**B**) Apoptosis assay by flow cytometry after treatment or not (control samples) to 62.5 µg/mL of onion extracts. (**C**) Relative cell cycle phases in ATDC-5 following a 24-h exposure to 62.5 g/mL onion extracts. (**D**) Quantification of ROS levels on ATDC-5 cells after 24 h incubation with onion extracts (1.9–62.5 µg/mL). Results are represented as fold change vs. control. Data are expressed as mean ± SD of at least three different assays carried out in triplicate and showed statistically significant differences between the control and the treated cells (* *p* ≤ 0.05; ** *p* ≤ 0.01; *** *p* ≤ 0.001; **** *p* ≤ 0.0001 vs. control). CTRL = control sample; OE = onion extract.

OE toxicity was evaluated in ATDC-5 cells exposed to different concentrations of extracts (1.9–62.5 µg/mL) for 24 h. Higher concentrations of these extracts reduced the viability to below 80% due to the quantity of DMSO added. The potentially harmful impact of OE on cell metabolism was evaluated using the Blue Cell Viability Assay. Cellular treatment with OE at increasing concentrations up to 62.5 μg/mL had no negative effects on the ATDC-5 cell line, yielding viability percentages higher than 90% (Figure 1A). These results are in agreement with previous studies in which different phenolic compounds did not exert cytotoxic effects in rabbits [39] and human chondrocytes [17]. Considering these results, further studies were performed at the highest OE concentration assayed (62.5 µg/mL).

Flow cytometry studies were carried out to evaluate the effects of OE on cell apoptosis and cell cycle phases. Figure 1B shows that apoptosis was not affected by OE treatment at the assayed concentration exhibiting similar necrosis, apoptosis, and viability percentages to untreated cells (control group), confirming the results obtained from the Blue Cell Viability Assay. However, cell cycle analysis showed that the S phase in OE-treated cells was increased (~15%) compared to control cells (Figure 1C). These data correlate with previous studies in which a similar trend was highlighted in rabbit chondrocytes proliferation after phenolic treatment [39].

Intracellular ROS level quantification was carried out in ATDC-5 cells at different OE concentrations (1.9–62.5 µg/mL) as depicted in Figure 1D. Intracellular ROS concentration slightly increased (10–30%) after OE treatment, which did not affect cell viability and apoptosis. This increase was not detrimental to ATDC-5 cells, thus, further experiments were carried out at the highest tested concentration (62.5 µg/mL). Though they may also have a pro-oxidant effect, phenolic compounds’ antioxidant activity has been extensively studied [31]. In addition to the cell line, phenolic plant compounds’ dual pro-oxidant and antioxidant action is also influenced by their concentration, chemical structure, and pH level [40,41].

#### 3.2.2. Molecular Changes after LPS and Onion Extracts Treatment

**Figure 2:**
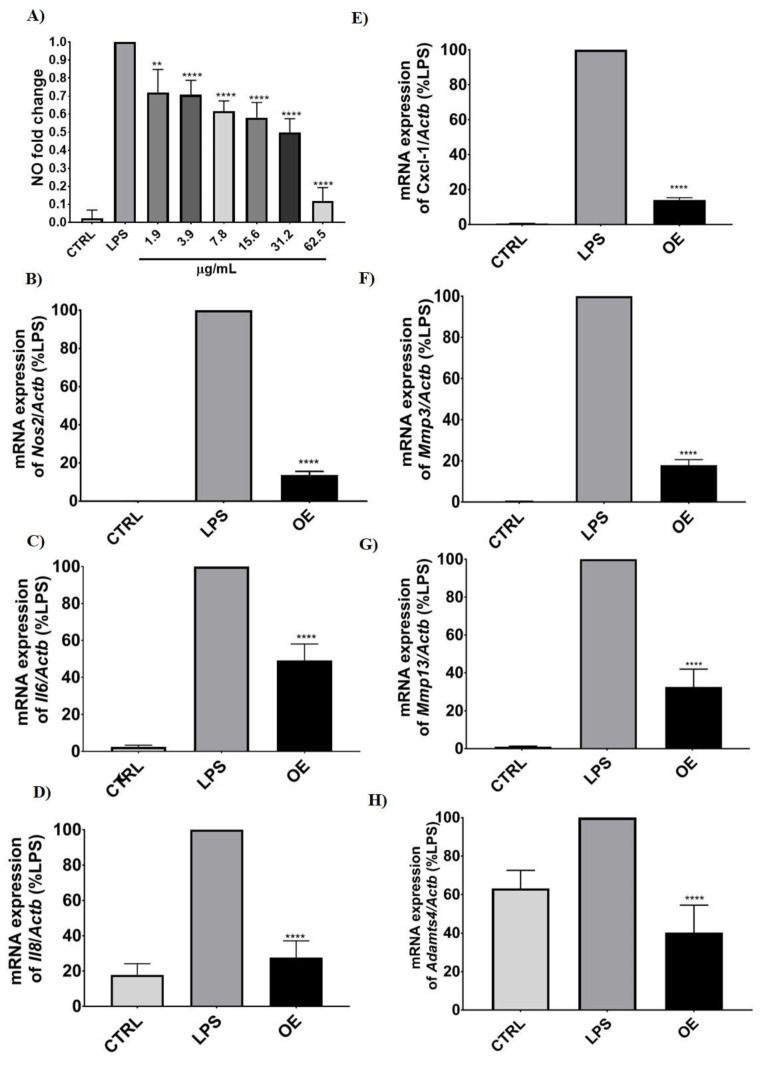
Onion extracts’ effects on LPS-induced NO generation. Results are expressed as a fold change vs. LPS (**A**) and expression of *Nos2* (**B**), *Il6* (**C**), *Il8* (**D**), *Cxcl1* (**E**), *Mmp3* (**F**), *Mmp13* (**G**), and *Adamtl4* (**H**) by RT-qPCR in ATDC-5 cell line. Cells were pretreated for 4 h with 62.5 g/mL of onion extract before being induced with LPS (250 ng/mL) or not (control cells) for 24 h. The level of the target mRNA was normalized to the level of *Actb* and expressed as a percentage of LPS, which was arbitrarily set to 100%. Data are expressed as mean ± SD of at least 3 independent experiments performed in triplicate and showed statistically significant differences between the control samples and the treated ones (* *p* ≤ 0.05; ** *p* ≤ 0.01; *** *p* ≤0.001; **** *p* ≤ 0.0001 vs. control). CTRL = control sample; OE = onion extract.

The anti-inflammatory ability of OE was studied in ATDC-5 cells treated with OE and then challenged with LPS. NO production, molecular expression of inflammation and cartilage damage markers, and NF-κB p65 translocation were studied. Figure 2A shows the efficacy of OE depleting NO production. NO levels significantly decreased following a concentration-dependent trend until the higher concentration assayed (62.5 µg/mL), at which NO production decreased by up to 90%. These results were confirmed at the molecular level, in which different inflammation and cartilage degradation markers were evaluated (Figure 2B–H). Expression of *Nos2*, *Mmp13*, *Mmp3*, *Il6*, *Il8*, *Cxcl1*, and *Adamtl-4*, which are relevant in cartilage damage, was carried out by RT-qPCR. The significant increase in the expression of these markers after cells received LPS treatment was depleted with the addition of OE. Control samples showed very low expression or even no expression of the genes evaluated. When cells were treated with LPS, their expression dramatically increased, except for the cartilage marker *Adamtl4,* in which a slight increase was found. The treatment with OE leads to the reversion of LPS-driven induction to an expression level similar to the control with a decrease higher than 50%. Specifically, the proinflammatory markers, *Nos2* and *Cxcl1*, together with the cartilage damage marker *Mmp3*, were the most effectively downregulated mRNAs, showing a decrease in their expression higher than 80%. These findings will also be further explored on the protein level.

**Figure 3:**
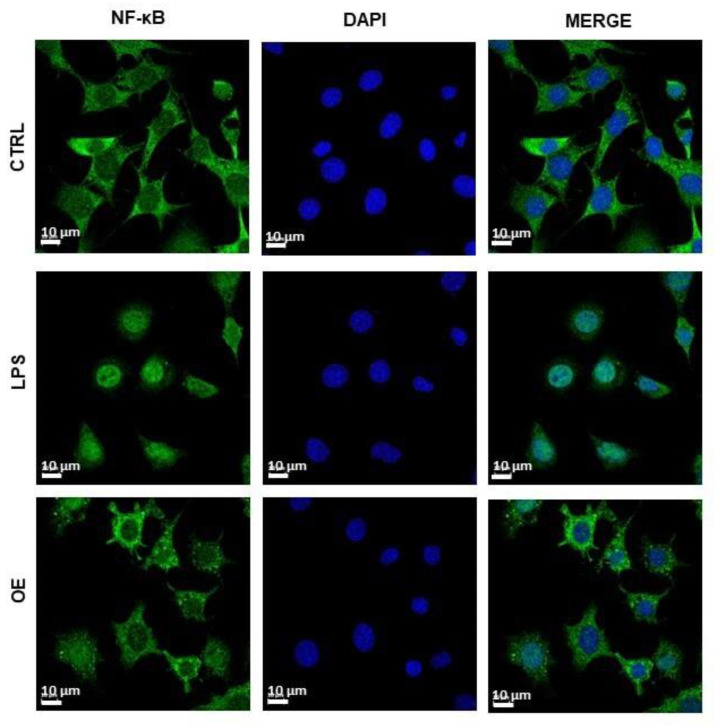
NF-B p65 nuclear translocation in ATDC-5 cell line induced by LPS and treated with onion extracts. Cells were not treated with LPS as CTRL sample (upper row), treated with LPS alone for 1 h (middle row), or co-treated with LPS and 62.5 µg/mL of onion extracts for 1 h (bottom row). NF-κB p65 was stained with Alexa Fluor^®^ 488 (green) while nuclei were stained with DAPI (blue). Scale bar = 10 µm. CTRL = control sample; OE = onion extract.

In this regard, nuclear translocation of NF-κB p65 induced by LPS in ATDC-5 cells was reverted by OE treatment (Figure 3). LPS treatment triggered the translocation of the p65 subunit of NF-κB to the nucleus pointing to the phosphorylation of the IKK complex and the activation of inflammatory pathways. The addition of OE (62.5 µg/mL) clearly decreased the translocation of p65 showing higher expression in the cytoplasm than in the nuclei. In this way, ROS may stimulate the NF-κB pathway in the cytoplasm while inhibiting it in the nuclei [42,43]. As a result, the NF-κB pathway in the cytoplasm may be activated by the increase in ROS levels (Figure 1D) caused by onion extract treatment.

These findings demonstrated the potential of OE to alleviate inflammation and chondrocyte catabolism, which are the main hallmarks of OA. Previous studies have pointed to the beneficial effects of phenolic compounds in inflammation and cartilage degradation. Human cartilage explants and chondrocyte cultures treated with these compounds obtained from Amazonian medicinal plants showed chondroprotective effects, reducing MMPs expression and ECM component release [15]. According to these studies, Lim et al. [10] evaluated the molecular changes in a human chondrosarcoma cell line treated with IL-1β and the subsequent treatment with different flavonoids (e.g., quercetin, kaempferol) at concentrations up to 25 µM. The expression of the cartilage catabolic marker *Mmp13* was significantly decreased, along with the inhibition of inflammation pathways. Quercetin and its chemical analogs were also demonstrated to be powerful disease-modifying compounds by diminishing inflammation and chondrocyte catabolism molecular markers [13,14]. Conversely, protocatechuic acid, another phenolic compound, has also shown chondroprotective potential ameliorating cartilage degradation in a long-term ex vivo porcine model [12] and in rabbit articular chondrocytes [39] at similar doses (25–50 µg/mL) as reported in the present study. In this line, linarin, a natural flavonoid glycoside, diminished several inflammatory markers, prevented chondrocyte catabolism and blocked NF-κB p65 activation in LPS-induced human OA chondrocytes [8]. Moreover, oxyresveratrol and phenolic compounds from *Morus alba* extracts showed a great potential to suppress inflammatory mediators and MMP-13 in IL-1 β- stimulated C28/I2 human chondrocytes [44]. The phenolic compound oleocanthal, found in extra virgin olive oil, also exerted anti-inflammatory and chondroprotective effects through MAPKs/NF-κB signaling in LPS-induced human OA chondrocytes [9].

### 3.3. Membrane Permeability and Pharmacokinetic Properties of Phenolic Compounds

There is no doubt that modern medicine faces one of its biggest challenges, the effective treatment of musculoskeletal disorders. In this regard, an effective strategy aiming to surpass these health problems must address the absorption, distribution, and metabolism of novel bioactive compounds [45]. In that sense, in silico analysis arises as a novel strategy that allows the computational simulation of the individual phenolic compound structures to address either their pharmacokinetic properties, biological activity, or toxicity. Thus, in silico analysis was added to this study to further understand the behavior and potential contribution to the anti-inflammatory and chondroprotective potential of OE (Table 1). The first parameter to discuss is the passive membrane permeability of phenolic compounds, which is shown as the apparent permeability (log P_app_). According to diverse studies, bioactive compounds are considered to have high permeability when they present a log P_app_ value higher than 0.90 × 10^−6^ cm/s [46,47]. In this regard, phenolic compounds from OE had moderate to low passive membrane permeability, as the highest value was observed for protocatechuic acid (0.49), followed by ellagic (0.335) and vanillic acid (0.33).

Another relevant parameter obtained from the in silico analysis is the distribution of phenolic compounds from OE, which is depicted as the steady state volume of distribution (VDss), blood–brain barrier (BBB) permeability, fraction unbound, and central nervous system (CNS) permeability. Regarding the VDss of the individual phenolic compounds from OE, it describes the volume of distribution during steady state conditions, such as when there is a stable drug concentration. According to the pkCSM tool, it could be considered as a low distribution when the log Vdss values are below −0.15 and a high good distribution volume when the log Vdss values are above >0.45. In that sense, flavonols are those compounds in OE with good distribution volumes, having the highest values quercetin 3-glucoside (1.846), followed by quercetin (1.559), kaempferol (1.274), and isorhamnetin (1.123). As for the BBB, those phenolic compounds with logBB lower than −1 are considered to be poorly distributed to the brain, while those with values higher than 0.3 are considered to cross the BBB. Thus, only protocatechuic acid could be considered to cross the BBB, as the others could be poorly distributed. Furthermore, the unbound fraction of the phenolic compounds from OE ranged from 0.083 (ellagic acid) to 0.648 (protocatechuic acid). As reported by Pratap et al. [48], the effectiveness of a drug may differ according to its capacity to interact or bind to blood proteins. Thus, those drugs that can more easily transverse the cellular membrane are those with a higher capacity to bind with blood proteins.

Finally, the metabolism of phenolic compounds from OE was assessed in terms of their role as inhibitors or substrates of CYP450 isoenzymes. In that sense, it was reported that nearly 8.9% of drugs metabolized in the liver are biotransformed by CYP1A2 [49]. That fact becomes highly relevant in this study, as the in silico analysis showed that ellagic acid, quercetin, and kaempferol from OE were CYP1A2 inhibitors. Similar results were reported by Al-Nour et al. [50], pointing to the potential decrease in the biotransformation of drugs, which probably increases the potential side effects. Nevertheless, it has been thoroughly studied that phenolic compounds promote human health due to their alleged beneficial effects against vascular and bone diseases such as atherosclerosis, osteoporosis, diabetes mellitus, and certain cancers. Therefore, the drug interactions with dietary phenolic components become highly relevant since these compounds can inhibit CYP450 isoenzymes. Specifically, Zendulka et al. [51] discussed that flavonoids are usually described as inhibitors of CYP450, and it is thought that the inhibition of CYP1A2 is part of their cancer preventive action. In that sense, it was suggested that the phenolic compound-induced inhibition of CYP1A2 prevents metabolic activation of procarcinogens, especially polycyclic aromatic hydrocarbons [52,53].

## 4. Conclusions

This work proves that onion waste is an important source of phenolic compounds that can be used in novel potential therapeutic approaches aimed to control inflammation as well as by controlling the oxidative status. Moreover, this study highlights the value of the extracts obtained from onion residues to be used as promising sources for functional food or nutraceutical products with anti-inflammatory and chondroprotective properties. In fact, these extracts, containing different bioactive compounds, may also exhibit synergistic effects that could enhance their therapeutic effects. In vitro studies showed that onion extracts had a high content of phenolic compounds and antioxidant properties. Likewise, cytocompatibility was demonstrated in the chondrogenic cell line ATDC-5, exerting viability percentages greater than 90% and a slight increase in the S phase of the cell cycle. Induction of inflammation mediated by LPS and treatment of cells with OE clearly demonstrated the reduction in NO production, as well as the inhibition of molecular markers related to inflammation and cartilage degradation. According to the in silico analyses, the in vitro effects observed could be related to the higher passive membrane permeability of phenolic acids, especially protocatechuic acid, though the use of these extracts should consider that molecules such as ellagic acid may inhibit CYP450 isoenzymes, changing the metabolism of certain drugs.

Therefore, this study provides new insights into the potential use of OE as functional ingredients with anti-inflammatory and chondroprotective properties in diseases such as OA. In addition, its use can contribute to the reduction in agro-food waste in the environment, facilitating the circular economy.

## Figures and Tables

**Table 1:** Pharmacokinetic properties of the phenolic compounds analyzed.

Pharmacokinetic Properties (Property; Units)	Protocatechuic Acid	Ellagic Acid	Vanillic Acid	Quercetin	Quercetin 3-Glucoside	Kaempferol	Isorhamnetin
Passive cellular permeability (Absorption; log Papp in 10–6 cm/s)	0.49	0.335	0.33	−0.229	0.242	0.032	−0.003
Water solubility (Absorption; log mol/L)	2.069	−3.181	−1.838	−2.925	−2.925	−3.04	−3
Human intestinal absorption (Absorption; % Absorbed)	71.174	86.684	78.152	77.207	47.999	74.29	76.014
Skin permeability (Absorption; log Kp)	−2.727	−2.735	−2.726	−2.735	−2.735	−2.735	−2.735
P-glycoprotein substrate (Absorption; Yes/No)	No	Yes	No	Yes	Yes	Yes	Yes
P-glycoprotein I inhibitor (Absorption; Yes/No)	No	No	No	No	No	No	No
P-glycoprotein II inhibitor (Absorption; Yes/No)	No	No	No	No	No	No	No
VDss (human) (Distribution; log L/kg)	−1.298	0.375	−1.739	1.559	1.846	1.274	1.123
Fraction unbound (human) (Distribution; Fu)	0.648	0.083	0.518	0.206	0.228	0.178	0.091
BBB permeability (Distribution; log BB)	0.683	−1.272	−0.38	−1.098	−1.688	−0.939	−1.135
CNS permeability (Distribution; log PS)	−3.305	−3.533	−2.628	−3.065	−4.093	−2.228	−3.188
CYP2D6 substrate (Metabolism; Yes/No)	No	No	No	No	No	No	No
CYP3A4 substrate (Metabolism; Yes/No)	No	No	No	No	No	No	No
CYP1A2 inhibitor (Metabolism; Yes/No)	No	Yes	No	Yes	No	Yes	Yes
CYP2C19 inhibitor (Metabolism; Yes/No)	No	No	No	No	No	No	No
CYP2C9 inhibitor (Metabolism; Yes/No)	No	No	No	No	No	No	No
CYP2D6 inhibitor (Metabolism; Yes/No)	No	No	No	No	No	No	No
CYP3A4 inhibitor (Metabolism; Yes/No)	No	No	No	No	No	No	No

## Data Availability

The data presented in this study are contained within the article.
